# New Insight into Laryngo-Tracheal Surgery: High-Flow Oxygen Therapy to Prevent Early Complications after Surgery

**DOI:** 10.3390/jpm14050456

**Published:** 2024-04-25

**Authors:** Beatrice Trabalza Marinucci, Silvia Fiorelli, Alessandra Siciliani, Cecilia Menna, Matteo Tiracorrendo, Domenico Massullo, Federico Venuta, Erino Angelo Rendina, Anna Maria Ciccone, Antonio D’Andrilli, Mohsen Ibrahim, Giulio Maurizi

**Affiliations:** 1 Thoracic Surgery, Sant’Andrea, Hospital, La Sapienza University, 00189 Rome, Italy; asiciliani@ospedalesantandrea.it (A.S.); cecilia.menna@uniroma1.it (C.M.); matteo.tiracorrendo@uniroma1.it (M.T.); erinoangelo.rendina@uniroma1.it (E.A.R.); annamaria.ciccone@ospedalesantandrea.it (A.M.C.); antonio.dandrilli@uniroma1.it (A.D.); mohsen.ibrahim@uniroma1.it (M.I.); giulio.maurizi@uniroma1.it (G.M.); 2 Anesthesiology and Intensive Care, Sant’Andrea Hospital, La Sapienza University, 00189 Rome, Italy; silvia.fiorelli@uniroma1.it (S.F.); dmassullo@ospedalesantandrea.it (D.M.); 3 Thoracic Surgery Policlinico Umberto I, La Sapienza University, 00186 Rome, Italy

**Keywords:** tracheal surgery, peri-operative management, high-flow oxygen therapy

## Abstract

Background: Early post-operative airway management after laryngo-tracheal surgery is crucial. Acute respiratory failure due to glottis’ edema may occur, requiring reintubation. This can prolong ventilatory assistance, jeopardizing anastomosis. To date, only judicious steroid administration and fluid management are available to avoid more invasive procedures. High-flow oxygen therapy (HFOT) is a noninvasive O_2_ support method providing humidification, warmed air, and Positive End-Expiratory Pressure (AIRVO2). No data about HFOT use to prevent early complications after laryngo-tracheal surgery are reported in the literature. Methods: Between September 2020 and September 2022, 107 consecutive patients who underwent laryngo-tracheal surgery received HFOT (Group A). Data and long-term results were compared with those of 80 patients operated between September 2018 and August 2020 (Group B), when HFOT was not available. All patients were operated in a single center. No pre- or post-operative settings changed, except for HFOT introduction. We analyzed and compared the risk for “delayed” reintubation (unexpected reintubation within the first 24–48 h after extubating/laryngeal mask removal) in the two groups. Results: No patients reported HFOT-related adverse events. The control group (B) presented “delayed” reintubation in 37% (*p* = 0.027), intensive care unit admission in 67% (*p* = 0.005) and longer hospital stay (*p* = 0.001) compared to the HFOT group (A). The minor complications’ rate was 3% in both group and overall mortality was 0%. Re-stenosis was described in 4.6% of the HFOT group, without a statistically significant difference (*p* = 0.7006). Conclusions: Our study is the first to investigate HFOT use in patients undergoing laryngo-tracheal surgery, potentially representing a consistent innovation in the peri-operative management of these patients. With the limitation of a retrospective series, we would suggest HFOT use for preventing post-operative reintubation rate, possibly reducing ICU admissions and hospital stays.

## 1. Introduction

Tracheal surgery for subglottic stenosis is a major therapeutic challenge. To date, single-stage resection with primary end-to-end anastomosis has proved to offer the best option of cure, allowing for a definitive and stable high success rate [1,2,3,4].

Nevertheless, post-operative complications may occur. Airway management in the immediate post-operative period following laryngotracheal surgery is of the utmost importance. Non-anastomotic complications include laryngeal edema and glottic dysfunction in phonation or swallowing [5]. Acute respiratory failure due to edema of the glottis could require reintubation or temporary tracheostomy in the immediate post-operative period [6,7]. This may prolong respiratory ventilatory assistance, increasing the rate of ICU admission, and the risk of anastomosis damage due to the cuff of the endotracheal tube lying on the mucosa or to mechanical ventilation’s pressures on the fresh anastomosis. 

To date, only judicious steroid administration, diuretics, nebulized epinephrine, and fluid management are available to prevent edema of the glottis in this set of patients [8,9].

Nowadays, several preventive strategies such as intra-operative protective mechanical ventilation, post-operative physiotherapy, and non-invasive mechanical ventilation (NIV) are available to reduce the incidence of early-post-operative complications in patients undergoing thoracic surgery [10]. Few and undersized studies have investigated the preventive role of HFOT (high-flow oxygen therapy, using a nasal cannula) after thoracic surgery, and there are no data regarding tracheal surgery [11,12]. The rationale of HFOT application is ensuring adequate oxygenation, increasing the clearance of secretions, and maintaining upper airway function and patency thanks to the moderate levels of generated Positive End-Expiratory Pressure (PEEP) [13,14]. In fact, acute respiratory failure due to edema of the glottis could require an unexpected re-intubation, defined in the present study as “delayed” re-intubation: the necessity to intubate patient post-surgery, after being extubated or after laryngeal mask removal. In contrast, we define “early re-intubation” as the necessity to put an endo-tracheal tube after the cross-field ventilation, during surgery, because of the intra-operative evidence of significant laryngeal edema that limits ventilatory assistance.

The aim of the present study is to investigate the role of HFOT in the prevention and treatment of intra- and post-operative laryngeal edema, thus avoiding unexpected re-intubation in patients undergoing tracheal resection-anastomosis for laryngo-tracheal stenosis. 

## 2. Materials and Methods

### 2.1. Ethical Statement

This retrospective cohort study was approved by the institutional review board (Prot. n. 21 SA/2023, RIF. CE 7063/2023) and it was conducted in accordance with the Declaration of Helsinki. Written informed consent was obtained from all patients and data were retrospectively analyzed. Consent for photo publication was obtained from the patient in the central picture.

### 2.2. Population

Between September 2018 and September 2022, 187 consecutive patients were treated for laryngo-tracheal stenosis in a single center. From September 2020, HFOT was introduced as a noninvasive O_2_ support method in the management of patients undergoing laryngo-tracheal resection-anastomosis, providing air humidification, warming, and PEEP (AIRVO2). The general population was divided into Group A (HFOT Group), patients treated between September 2020 and September 2022 (*n* = 107), and Group B (control Group), patients treated between September 2018 and August 2020 (*n* = 80). The patients in Group A received post-operative HFOT and it was applied after extubating/laryngeal mask removal for the first 48 h after surgery if no adverse events were referred by the patient. The patients in Group B did not receive HFOT. We included in the study all patients affected by benign laryngo-tracheal stenosis and fit for surgery. The exclusion criteria were as follows: (i) malignant stenosis, (ii) patients unfit for surgery (low-performance status; severely impaired cardiac function; NYHA IV; important neurologic disorders severely limiting or abolishing patients’ cooperation), and (iii) stenosis limited to medial-cervical and thoracic trachea.

### 2.3. Pre-Operative Assessment

Preoperative assessment included fiberoptic bronchoscopy (FBO) to verify the vocal cords status (motility and integrity), and characteristics of the glottis and the stenosis (distance from vocal folds, extent, and severity grade according to Meyer–Cotton classification). Also, a neck and thorax computed tomography (CT) scan was used to better evaluate the tracheal wall, distance from vocal cords, and tissue status (using medium contrast). 

Tracheal surgery was performed by a specialized team of experienced surgeons and anesthesiologists who have many years of training. Every member of the medical team applied the same standardized operative methods over the years of the study. 

### 2.4. Intra-Operative Management

Intra-operative ventilation was achieved by endotracheal intubation with a wire-reinforced small caliber tube (size 4–6.5 mm) passed through the stenosis or by the insertion of a laryngeal mask airway (LMA) device (size 4–5) to avoid dilatation at the time of intubation and consequent trauma. The choice between endo-tracheal tube (ETT) and laryngeal mask was carried out according to the patients’ physical characteristics, type of stenosis (distance from vocal cords, grade of stenosis), and clinical history (Obstructive Syndrome Apnea Syndrome, previous radiotherapy, or previous laryngeal procedure, pre-operative laryngeal edema, critical stenosis, and obesity contraindicate LMA use). When LMA was introduced, it was preferentially chosen if not contraindicated. 

Laryngo-tracheal resection-anastomosis was performed in accordance with Pearson’s technique: Through a collar cervical incision, the cervical trachea is exposed and circumferentially mobilized from the inferior border of cricoid cartilage to the lower limit of the stenosis; then, the distal trachea is transected and intubated through the operative field (cross field-intubation). The upper line of resection continues from the lower limit of the cricoid to the crico-thyroid joints, on both sides, and then the anterior arch of the cricoid is transected, leaving the posterior plate. The reconstruction was performed by an end-to-end anastomosis with interrupted suture on the anterior wall and continuous suture on the posterior wall using an absorbable monofilament (polydioxanone). A personal variation (partial laryngo-fissure) when vocal cords were involved is described in detail in previous work [15] to obtain an enlargement in the antero-posterior diameter of the airway. 

During surgery, all patients underwent traditional cross-field ventilation after tracheal transection by an armored 5 mm endo-tracheal tube (ETT). The two groups received the same intra-operative anesthetic management, using fentanyl 2 mcg/kg, propofol 2 mg/kg, i.v., and rocuronium 0.8 mg/kg. 

Intra-operative edema is defined as reduction in the arytenoid and vocal cords’ motility, an augmented width of vocal cords, and a reduction in glottis space. It is assessed through bronchoscopy, and classified according to the Laryngeal Edema Classification (LOC) [16]: LOC-1 (25% obstruction of the supra-larynx, few symptoms), LOC-2 (50% obstruction of the supra-larynx, moderate symptoms), LOC-3 (75% obstruction of the supra-larynx, severe symptoms), and LOC-4 (90% obstruction of the supra-larynx, acute threat of life). Post-extubation and/or laryngeal mask removal edema were clinically evaluated and defined as ”Minor” (presence of stridor; that is to say, an audible high-pitched inspiratory wheeze and signs of respiratory distress, i.e., a prolonged inspiratory phase with recruitment of accessory respiratory muscles as seen by subcostal, suprasternal or intercostal retraction) and ”Major” (respiratory distress needing tracheal intubation secondary to upper airway obstruction), and then confirmed by fiberoptic-bronchoscopy. [17]

### 2.5. Post-Operative Management

Extubation in the operating room was tried for every patient when possible. Patients with intra-operative evidence of laryngeal edema received traditional endotracheal intubation after the cross-field ventilation with a naso-tracheal tube (defined in the present study as “early re-intubation”, re-intubation during surgery, in contrast with post-surgery delayed re-intubation). From September 2020, patients started HFOT directly in the operating room after being successfully extubated. When extubation failed, a naso-tracheal tube was positioned as described in our previous study, with a 7–7.5 mm caliber ETT with a deflated cuff. This unexpected re-intubation is defined as “delayed re-intubation”, occurring within the first 24–48 h of surgery (in contrast with early re-intubation). Since we increased the use of LMA, default intubation post-surgery is avoided and an intra-operative fiberoptic check is routinely performed to check the glottis’ status: if intra-operative laryngeal edema is observed, there is the indication to proceed to early re-intubation, leaving an ETT after surgery. Each member of the team applied the same described evaluation criteria to make a decision regarding the patients’ re-intubation. 

No step down or progressive care units are available in our center. The need for post-operative multiparametric monitoring in ICU was based on the individual evaluation of the patient, total operative time, patient stability and cooperation, and early re-intubation. 

In Group A, HFOT was used for the first 48 h after surgery in addition to classical management: steroids (Metilprednisone i.v. 10 mg, once); fluid management (1.5 lt i.v. and/or orally when the patient re-start feeding); and diuretics (Furosemide i.v. 25.0 mg, twice). Patients receiving HFOT did not have air humidification in the room nor nebulizers (epinephrine nebulizer is recommended just in case of stridor). Patients who did not receive HFOT received traditional non-invasive methods (steroid administration, air humidification, diuretics therapy, nebulized epinephrine) to prevent and to threat laryngeal edema. No other settings in the peri-operative management have changed over the years. When applied, HFOT was routinely set at 40 L/min of oxygen flow, with 40% of FiO_2_, PEEP 2–3 mmHg, and a temperature of humidification 37 °C to maintain a peripheral oxygen saturation (sP02) ≥ 95%. Arterial blood gases (ABG) and sP02 were controlled after 1 h, 6 h, and 12 h. Personal discomfort and HFOT adverse events (throat or nasal pain, abdominal distension) self-reported by the patients were registered [18]. 

During the following hospital stay, FBO was carried out on post-operative day 1 for the re-intubated patients and at discharge for every patient. 

### 2.6. Statistical Analysis

Data were collected and stored in an Excel database (Microsoft Corp, Redmond, WA, USA) and were analyzed using statistical package SPSS, version 25.0 (SPSS Software, IBM Corp., Armonk, NY, USA). Quantitative variables were expressed as mean ± standard deviation, whereas nominal variables were expressed binarily as presence (1) or absence (0) of the event. A comparison of the categorical variables was performed by the c2 test using Fischer’s exact test. A comparison of continuous variables was performed by Student’s *t*-test. Significance was defined as a *p*-value of less than 0.05. A univariable logistic regression analysis was performed to derive crude estimates of association between predictors (predictive variables defined as “independent variable” and outcomes (defined as “dependent variable”, such as intra- and post-operative laryngeal edema), minimizing potential confounders (exclusion criteria: release, neoplastic, and carinal stenosis). After univariable analyses, variables with a *p*-value of less than 0.05 were included in a multivariable logistic regression model to identify potential independent protective or risk factors. The adjusted odd ratios (ORs) and 95% confidence intervals (CI) were calculated to estimate and measure the association using 1000 bootstrapping samples.

## 3. Results

Male patients were 20/107 (19%) in the HFOT group (Group A) and 25/80 (31%) in the control group (Group B); the average age of population was 56.11 ± 4.34 in Group A and 54.21 ± 3.75 in Group B. In Group A, 89/107 (83%) patients were operated with laryngeal mask intra-operative assistance, whereas 18/107 (17%) patients were operated with traditional endotracheal tube. In Group B, 46/80 (58%) patients were operated with laryngeal mask, and 34/107 (42%) were operated with ETT. 

In Group A, 2/107 (1.9%) patients underwent laryngo-fissure vs. 4/80 (5%) in Group B, without a statistically significant difference (*p* = 0.4127). 

The general characteristics are described in Table 1.

The mean operative time was not different in the HFOT group (76.50 ± 28.48 min) compared to the control group (77.20 ± 25.51), while ICU admission was higher in Group B (*n* = 54/80, 67%) than in Group A (*n* = 50/107, 47%), with a statistically significant difference, *p* = 0.005. The mean time of ICU stay was not statistically different between Group A (0–5; 3 ± 2.3 days) and Group B (0–7; 4 ± 4.2 days), but the mean time of hospitalization was longer in Group B (7 ± 3.7 days) than in Group A (5 ± 2.5 days), *p* = 0.0010.

The “delayed re-intubation” rate was higher in control group (30/80; 37%) compared to that in the HFOT group (25/107; 23%), with a statistically significant difference, *p* = 0.027. In the HFOT group, 8/107 (9%) patients operated with laryngeal mask needed delayed reintubation vs. 5/80 (10%) in Group B. 

Mortality was 0% in both groups and minor complications (vocal roughness, initial swallowing difficulty, and inaesthetic scar) were 3% in both groups. 

One patient in Group A (0.9%) received temporary tracheostomy because of vocal cord paralysis vs. one patient in Group B, without a statistically significant difference (*p* = 1). Only 2/80 (2.5%) patients in the control group experienced re-stenosis (one of them after re-intubation) at the follow-up compared to 5/107 (4.6%) patients in the HFOT group (no one of these experienced re-intubated), without a statistically significant difference (*p* = 0.7006).

Univariable logistic regression was applied to identify the independent variables (among age, sex, idiopathic stenosis, Meyer–Cotton stage, presence of intra-operative edema, vocal cords distance, and HFOT application) related to post-operative laryngeal edema: idiopathic stenosis (*p* = 0.001) and HFOT (*p* = 0.004) were found to be significatively related to post-operative laryngeal edema, and the multivariable analysis confirmed idiopathic stenosis (*p* = 0.001) as a risk factor and HFOT (*p* = 0.010) as a protective factor for post-operative laryngeal edema (Table 2).

## 4. Discussion

Laryngeal edema is one of the most challenging intra- and post-operative non-anastomotic complications following laryngo-tracheal surgery, eventually requiring the patient’s re-intubation. 

Despite the possible complications, many authors concluded that primary resection-anastomosis is the gold standard for the treatment of tracheal and/or laryngo-tracheal stenosis: Grillo et al. [19] reported a 91% rate of good-to-excellent outcomes over a series of 35 single-staged laryngotracheal resections for idiopathic stenosis; Ashiku et al. [20] observed the same rate (91%) of good-to-excellent long-term results; Morcillo et al. [21], in a Spanish multi-institutional study, reported a 97% final success rate; and finally, Maurizi et al. reported a series with a definitive success rate of 98.7% [17]. The Society of Thoracic Surgeons General Thoracic Surgery Database (STS GTSD) analyzed 1,617 patients undergoing tracheal resection between 2002 and 2016 and reported an overall 30-day mortality of 1% [22].

Post-operative care for patients after tracheal surgery includes judicious use of steroids, ambient humidification, and diuretics to prevent the risk for edema of the glottis, eventually causing respiratory failure and the need for the patient’s re-intubation. Recently, high-flow oxygen therapy using a nasal cannula (HFOT), a system capable of delivering a high flow (30–60 L/min) of heated and humidified gas at a controlled oxygen concentration, has become increasingly used in the prevention and treatment of post-extubation respiratory failure in surgical patients [23]. Several studies have shown that HFOT reduces post-operative hospital stay, ICU readmission, risk of intubation in acute hypoxic respiratory failure, and it increases respiratory support compared with traditional oxygen administration. HFOT humidification and warming of inspired gas guarantees secretions’ dilution, enhancing mucociliary clearance and expectoration [24].

In addition, HFOT results in a positive end-expiratory pressure (PEEP), reaching values of 5–6 cmH_2_O, ensuring airway patency. 

**Figure 1:**
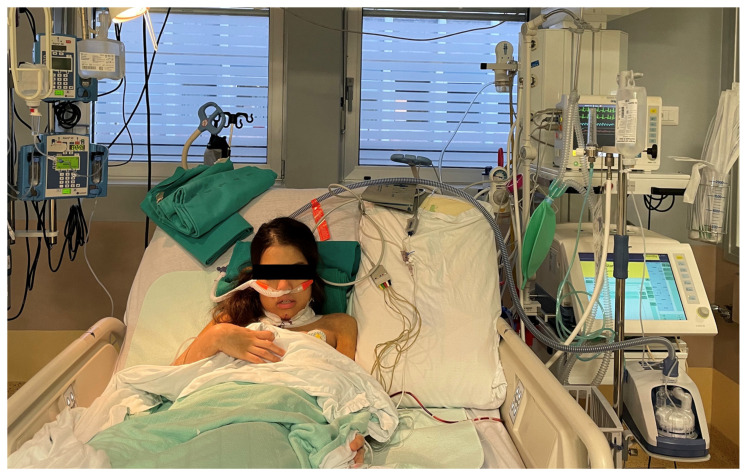
Patient with high-flow oxygen therapy support (AIRVO2 machine) immediately after-tracheal resection for subglottic stenosis (original picture; consent obtained for photo publication).

To the best of our knowledge, HFOT in tracheal surgery has not yet been reported and the present study is the first investigating the potential role of HFOT in the prevention and treatment of laryngeal edema in patients undergoing laryngo-tracheal surgery (Figure 1).

HFOT in tracheal surgery takes advantage of the airways’ warming and humidification to guarantee mucosal integrity and physiology, and, mainly, it takes advantage of the PEEP generated that may contribute to keep the upper airways open, thus contrasting laryngeal edema [25,26,27].

The oxygen inflow was set at the minimum because its application was neither for a hypoxic problem nor for hyper-carbic respiratory failure. In fact, the rationale was no longer oxygen administration (also useful to mucosal integrity) but the dilution of secretions due to the direct flow of humidification, and the patency of the upper airway, guaranteed by the minimal PEEP [28,29]. In fact, the main observed effect is the reduction in the secretions’ density, allowing for their easier expectoration, thus reducing the risk of mucous plugs that can cause temporary desaturation and a sense of breathiness and discomfort [30]. Moreover, HFOT allows for an enhanced recovery after surgery because patients can move from the bed, only wearing the nasal canula, and they are not bedside-constricted because of the necessity for continuous traditional air humidification. 

The present study reports a significative reduction in the rate of ICU admission (*p* = 0.005) in patients who underwent HFOT compared to the control group. Similarly, the incidence of delayed re-intubation was lower in Group A: in the uni- and multivariable analyses, HFOT was identified as a protective factor for post-operative laryngeal edema, thus probably preventing unexpected re-intubation due to post-operative edema of the glottis. 

No difference was noticed in ICU stays, but patients who did not receive HFOT (Group B) had a higher rate of access to ICU and a longer hospitalization, in accordance with the higher rate of delayed re-intubation described in this group. In fact, ICU is not necessary for every patient, and it is avoided, if possible, to enhance early recovery after surgery. Probably, the higher rate of ICU admission in Group B is also related to the most frequent use of ETT in this group (42% of patients operated on with ETT in Group B vs. 17% in Group A; *p* = 0.0001), left on site after surgery. In fact, patients operated with LMA generally do not require nasal–tracheal tube positioning if intra-operative edema does not occur, reducing ICU admissions. ICU length of stay has a standard length of stay of 12–24 h in both groups, but it must be considered that unexpected re-intubation occurs in the first 12–24 h after extubation/laryngeal mask removal; so, in the end, many days of hospitalization added up in Group B independently of the single ICU length of stay. Moreover, patients who underwent delayed re-intubation required precautionary wait-and-see management and more conservative approaches using standard methods (nebulizers, diuretics, rest, FBO controls) before discharge, increasing the total length of the hospital stay.

The present work confirms the most frequent incidence of laryngeal edema in very high subglottic stenosis (*p* < 0.001) and idiopathic stenosis (which is very high subglottic stenosis), and these represent an independent negative prognostic factor for post-operative laryngeal edema, eventually requiring re-intubation. In fact, the closer the stenosis is to the vocal cords, the greater the risk for inflammation involving vocal cords, and a consequent edema with respiratory failure and the necessity for re-intubation may occur in these cases [31]. Moreover, we found a significant difference in the patients’ sex: in recent years, more female patients underwent laryngo-tracheal resection in our unit, and these data are probably related to the well-known most frequent incidence of idiopathic stenosis in the female sex. 

Since we have started using LMA, the gold standard has been extubation in the operating room; so, the need for the use of preventive ETT for the first 24 h (what was carried out in the past) has been avoided when possible [9].

The use of laryngeal mask is less associated with post-operative laryngeal edema compared to ETT (*p* = 0.0001): only 9–10% of patients operated on with a laryngeal mask needed “delayed” re-intubation (after laryngeal mask removal). These results are in agreement with a previously published study by Menna et al. [32] supporting the hypothesis that LMA assistance reduces the risk of stenosis manipulation. Moreover, LMA allows for an intra-operative assessment of vocal cords status. So, LMA has certainly improved peri-operative management and the consequent outcomes, but its use does not totally avoid laryngeal edema, as previously described in the literature [28]. So, we do not think that the improvements described in recent years are linked to a single factor, but, probably, the association of both LMA and HFOT is advantageous to achieve increased post-surgical outcomes compared to the past. Previous studies reported that hyperbaric oxygen treatment contributes to the healing of tracheal anastomosis and may be a useful supportive treatment after tracheal resection and end-to-end anastomosis [33]. 

The increased rate of delayed re-intubation presented in this study with respect to our previously published study [15] is probably related to a higher incidence of idiopathic stenosis in recent years and to the increased age of patients; in fact, we observed a significant difference (*p* < 0.001) in age between the two groups, with a higher age of patients treated in Group A.

HFOT represents a simple and safe method, with low cost and easy applicability in routine post-operative clinical practice, causing little or no discomfort, as self-reported by patients that experienced no adverse effects related to its use (such as throat or nasal pain, or abdominal distension). 

Moreover, we found that the impact of reintubation on long-term airway patency was not statistically different in the groups: only two patients operated on between September 2018 and August 2020 experienced re-stenosis (one of them after re-intubation), while five patients operated between September 2020 and September 2022 experienced re-stenosis (none of them received re-intubation), without a statistically significant difference (*p* = 0.7006). So, re-intubation does not seem to affect the re-stenosis rate in the present study.

Our study has some limitations. Despite the high volume of patients, it is a single-center retrospective study; it deals only with early-post-operative complications (does not have a long time of follow-up); and in recent years, the complexity of the pathology has increased (augmented age of surgery, idiopathic subglottic stenosis), as well as the use of laryngeal mask, identifying some possible selection bias. Nevertheless, the population appears homogeneous and our preliminary results on HFOT after laryngo-tracheal resection-anastomosis are promising.

## 5. Conclusions

The authors’ research focuses on the initial examination of HFOT utilization in individuals who underwent laryngo-tracheal surgeries, possibly introducing a significant advancement in the care of these patients before and after their procedure. In conclusion, the results of this study suggest that the use of HFOT could be recommended as post-operative support in patients undergoing laryngo-tracheal resection-anastomosis, reducing the risk for delayed re-intubation due to post-operative laryngeal edema, and possibly reducing ICU admissions and hospital stays. 

Further prospective multicentric studies would be useful to confirm the promising results demonstrated by the present retrospective cohort study. 

## Figures and Tables

**Table 1:** Patient with high-flow oxygen therapy support (AIRVO2 machine) immediately after laryngo-tracheal resection for subglottic stenosis (original picture; consent obtained for photo publication).

Parameter	HFOT Group A (107/187)	Control Group B (80/187)	*p*
**Male Gender, n (%)**	20 (19%)	25 (31%)	0.035
**Age (years, mean ± SD)**	56.11 ± 4.34	54.21 ± 3.75	0.0097
**BMI (kg/m^2^, mean ± SD)**	24.57 ± 2.44	25.21 ± 1.97	0.0158
**Operative time (min, mean ± SD)**	76.50 ± 28.48	77.20 ± 25.51	0.4235
**Type of operation, n (%)**			
**Laryngo-fissure**	2 (1.9%)	4 (5%)	0.4127
**Pearson’s technique**	105 (98.1%)	76 (95%)	
**Stenosis distance from vocal folds (mm, mean ± SD)**	1.42 ± 0.78	1.52 ± 1.20	0.2368
**Intra-operative assistence n (%)**			
**LMA**	89 (83%)	46 (58%)	0.0001
**ETT**	18 (17%)	34 (42%)	
**ICU admission, n (%)**	50 (47%)	54 (67%)	0.005
**Delayed reintubation, n (%)**	25 (23%)	30 (37%)	0.027
**HFOT duration (h, mean ± SD)**	48 ± 12.42		
**HFOT flow (l/min, mean ± SD)**	38.5 ± 7.8		
**HFOT FiO_2_ (%, mean ± SD)**	36.5 ± 10.01		
**Discomfort during HFOT, n (%)**	20 (%)		
**Temporary tracheostomy, n (%)**	1 (0.9%)	1 (1.2%)	1.000
**ICU stay in days (range, mean ± SD)**	0–5, 3 ± 2.3	0–7, 4 ± 4.2	0.2610
**Hospital stay (days, mean ± SD)**	5 ± 2.5	7 ± 3.7	0.0010
**Re-stenosis, n (%)**	5 (4.6%)	2 (2.5%)	0.7006
**Mortality, n (%)**	0 (0%)	0 (0%)	1.0000

BMI: Body Mass Index; HFOT: High-flow oxygen therapy; ICU: intensive care unit; LMA: Laryngeal mask assistance; ETT: endo-tracheal tube; delayed re-intubation: re-intubation after post-surgery extubation or laryngeal mask removal.

**Table 2:** Multivariable logistic regression for post-operative laryngeal edema.

Variables	OR	*p* Value	95% CI
Idiopathic stenosis	3.859	0.001	1.946–7.650
HFOT application	0.409	0.010	0.206–0.810

HFOT: High-flow oxygen therapy.

## Data Availability

The original contributions presented in this study are included in this article; further inquiries can be directed to the corresponding author.
